# Multiscale computational genomics in Wilson disease: from atomic dynamics to clinical prediction

**DOI:** 10.3389/fgene.2026.1766223

**Published:** 2026-03-03

**Authors:** Moujun Luan, Qingkai Xue, Yujie Cao, Gangli Cheng, Xingxing Huo

**Affiliations:** Experimental Center of Clinical Research, The First Affiliated Hospital of Anhui University of Chinese Medicine, Hefei, Anhui, China

**Keywords:** ATP7B, computational genomics, machinelearning, molecular dynamics, multi-omics, precision medicine, systems biology, wilson disease

## Abstract

Wilson disease (WD) is an autosomal recessive disorder caused by pathogenic variants in the ATP7B gene, leading to toxic copper accumulation. The integration of computational genomics approaches is now essential for deciphering the complex genotype-phenotype relationships and advancing towards targeted therapies. This review synthesizes how multiscale computational strategies are transforming WD research. At the atomic level, molecular dynamics (MD) simulations reveal the conformational dynamics of the ATP7B protein, the functional impact of mutations, and the detailed copper transport cycle. At the systems level, machine learning (ML) models integrate genomic, epigenomic, transcriptomic, and clinical data to classify variant pathogenicity, predict disease subtypes, and forecast clinical outcomes such as cirrhosis or neurological deterioration. Furthermore, multi-omics network analyses uncover disease-associated regulatory modules, elucidate the role of epigenetic dysregulation, and implicate emerging pathways like cuproptosis in WD pathogenesis. Critically, these computational insights are increasingly guiding therapeutic innovation, including the in silico design of allosteric modulators (e.g., nanobodies) and pharmacological chaperones to correct ATP7B folding. By bridging scales from molecular structure to patient phenotypes, computational genomics provides a powerful, integrative framework that holds the potential to accelerate the development of dynamic, mechanism-based therapies and pave the way for personalized medicine in Wilson disease.

## 1 Introduction

Wilson Disease (WD), an autosomal recessive disorder driven by pathogenic ATP7B mutations that impair copper homeostasis, presents a major therapeutic challenge in hepatology and neurology (Dev et al., 2022). Clinically, it manifests with severe hepatic complications (e.g., cirrhosis, acute liver failure) and progressive neurological deterioration (including tremors, dystonia, and psychiatric disturbances) (Dev et al., 2022; Moini et al., 2021; Henderson et al., 2020). Diagnosis is frequently delayed due to phenotypic variability and the limited specificity of conventional biomarkers such as serum ceruloplasmin (Schilsky et al., 2025). Current first-line therapies—copper chelators (e.g., D-penicillamine) and zinc salts—are burdened by significant adverse effects and exhibit treatment failure in approximately 30% of cases, underscoring the urgent need for mechanistically informed interventions (Ganaraja et al., 2025; Aboalam et al., 2025; Roy et al., 2025). The pathogenesis of WD is fundamentally underpinned by dysfunctional ATP7B, a P-type ATPase that coordinates cellular copper export through Golgi-mediated trafficking and canalicular excretion (Pantoom et al., 2021; Ariöz et al., 2017). Recurrent pathogenic mutations (notably H1069Q and R778L) disrupt this protein’s copper transport capacity, folding stability, or subcellular trafficking, culminating in cytotoxic copper accumulation (Wang Y. et al., 2025; Zhang et al., 2022). Elucidating the structure-dynamics-function relationships of ATP7B is indispensable for developing targeted therapeutic strategies (Teschke and Eickhoff, 2024; Sánchez-Monteagudo et al., 2024).

Traditional approaches face profound challenges in deciphering mechanisms of WD (Vardhini et al., 2025). Structural techniques (cryo-EM, crystallography) capture static snapshots but miss rapid conformational transitions essential for copper transport (Liu et al., 2018; Ma et al., 2026). Functional assays struggle to resolve the allosteric effects of >800 pathogenic variants or the epistatic influence of genetic modifiers (Sarode et al., 2021). Moreover, WD’s phenotypic heterogeneity—driven by epigenetic dysregulation, metabolic disturbances, and multi-organ crosstalk—defies reductionist methodologies (Stalke et al., 2023; Wang et al., 2021). Computational biology offers a unified framework to bridge these distinct scales: Molecular dynamics (MD) simulations decode the structural consequences of mutations—such as destabilization—which serve as the mechanistic basis for the pathogenicity features used in Machine Learning (ML) models. Concurrently, network analyses contextualize these molecular defects within systemic regulatory hierarchies, explaining how copper homeostasis disturbances trigger downstream cell-fate decisions like cuproptosis (Dev et al., 2022; Lazim et al., 2020; Lutsenko et al., 2025). These approaches collectively transform static data into dynamic, mechanism-based insights (Bajwa et al., 2021; Rajpurkar et al., 2022; Ayub et al., 2025).

**FIGURE 1:**
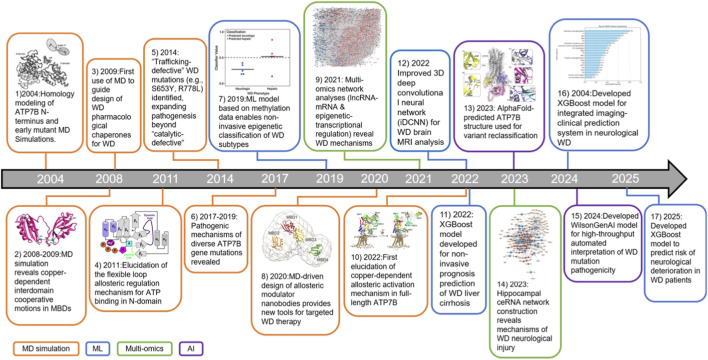
Timeline of the evolution of major advancements in computational biology for WD research. The field has progressed from atomic-level MD simulations of ATP7B towards ML for clinical prediction, multi-omics integration, and AI-driven structure prediction and therapeutic design, collectively enabling a path to precision medicine. See below for publication details corresponding to each entry. 1. 2004:Homology modeling of ATP7B N-terminus and early mutant MD Simulations (Efremov et al., 2004). 2. 2008–2009:MD simulation reveals copper-dependent interdomain cooperative motions in MBDs (Rodriguez-Granillo et al., 2009; Rodriguez-Granillo et al., 2010). 3. 2009:First use of MD to guide design of WD pharmacological chaperones for WD (van den Berghe et al., 2009). 4. 2011:Elucidation of the flexible loop allosteric regulation mechanism for ATP binding in N-domain (Hercend et al., 2011).5. 2014: “Trafficking-defective” WD mutations (e.g., S653Y, R778L) identified, expanding pathogenesis beyond “catalytic-defective” (Braiterman et al., 2014). 6. 2017–2019: Pathogenic mechanisms of diverse ATP7B gene mutations revealed (Yu et al., 2018; Kumar et al., 2017; Shanmugavel et al., 2019; McCann et al., 2019). 7. 2019:ML model based on methylation data enables non-invasive epigenetic classification of WD subtypes (Mordaunt et al., 2019). 8. 2020:MD-driven design of allosteric modulator nanobodies provides new tools for targeted WD therapy (Uhlemann et al., 2020).9. 2021: Multi-omics network analyses (lncRNA-mRNA and epigenetic-transcriptional regulation) reveal WD mechanisms (Sarode et al., 2021; Zhang et al., 2021). 10. 2022:First elucidation of copper-dependent allosteric activation mechanism in full-length ATP7B (Orädd et al., 2022). 11. 2022: XGBoost model developed for non-invasive prognosis prediction of WD liver cirrhosis (Chen et al., 2022). 12. 2022 Improved 3D deep convolutional neural network (iDCNN) for WD brain MRI analysis (Agarwal et al., 2021).13. 2023: AlphaFold-predicted ATP7B structure used for variant reclassification (Stalke et al., 2023). 14. 2023: Hippocampal ceRNA network construction reveals mechanisms of WD neurological injury (Wang et al., 2023). 15. 2024:Developed WilsonGenAI model for high-throughput automated interpretation of WD mutation pathogenicity (Vatsyayan et al., 2024). 16. 2004:Developed XGBoost model for integrated imaging-clinical prediction system in neurological WD (Yang et al., 2024). 17. 2025:Developed XGBoost model to predict risk of neurological deterioration in WD patients (Wang et al., 2025c).

This review adopts a multi-scale perspective to synthesize these advancements (Figure 1). (Dev et al., 2022; Zhao et al., 2024). We begin by detailing how MD simulations elucidate the atomic ‘cause’ of dysfunction, including allostery and misfolding (Section 3). Building on this mechanistic foundation, we next explore how data-driven frameworks—such as ML classifiers and epigenetic networks—translate these molecular features into clinical outcome predictions (Section 4). Finally, closing the loop between mechanism and therapy, we discuss how multi-omics integration guides the design of precision interventions, such as pharmacological chaperones that specifically target the folding defects identified *in silico* (Section 5). By converging these insights, this review proposes an integrative roadmap to accelerate the transition from mechanism discovery to personalized medicine in WD.

## 2 ATP7B protein: structural dynamics, functional mechanisms, and computational targeting

### 2.1 Domain architecture: from static folds to dynamic regulation

ATP7B, a central molecule in copper homeostasis, is fundamental to the precise coupling of its three-dimensional structure and function for copper ion transmembrane transport. The protein consists of three main structural modules: the N-terminal metal-binding domains (MBDs), the transmembrane domain (TMD), and the C-terminal catalytic domain (comprising the N-domain, P-domain, and A-domain) (Yang et al., 2023). The six tandem MBDs (MBD1-MBD6) at the N-terminus specifically bind Cu(I) via conserved MTCxxC motifs, forming the initial copper ion capture interface (Ariöz et al., 2017). For example, the CXXC motif (C575) in MBD6 interacts with methionine 729 (M729) in the transmembrane helix M1, facilitating copper ion transfer to the entrance of the transmembrane channel (Yang et al., 2023; Al-Obaidi and Al-Musawi, 2025). Cryo-EM structures reveal that MBDs dynamically couple with the TMD through flexible linkers. Upon copper binding, MBD6 undergoes conformational rotation and pulls transmembrane helices toward the cytosol, forming an ion-conductive pathway (Yu et al., 2018; Orädd et al., 2022).

The TMD, composed of eight α-helices, forms an inverted conical channel where key cytosolic residues coordinate Cu(I), and extracellular structural elements regulate ion release (Yang et al., 2023). Structural analyses highlight that transmembrane helices MB and M1-M2 create a cytosol-facing cavity, serving as the critical portal for copper ion entry. Upon ion entry through the MBDs-TMD interface, hydrophobic interactions within the TMD trigger helical rearrangements that open the channel exit (Concilli et al., 2020). Pathogenic mutations, such as the H1069Q mutation disrupt the coordination bond between His1069 and Cu(I), constricting the channel entrance and significantly prolonging copper ion transmembrane transport (Concilli et al., 2020). Additionally, the conserved CPC motif (C983-C985) in the TMD plays a pivotal role in copper transfer; its disruption leads to significant reductions in transport efficiency (Yang et al., 2023; Li M. et al., 2021).

The C-terminal catalytic domain drives conformational cycling via ATP hydrolysis: the N-domain binds ATP through a conserved motif, while the P-domain harbors the phosphorylation site Asp1027, linking nucleotide binding to conformational energy transfer (Xue et al., 2023). NMR spectroscopy highlights the N-domain’s structural homology to bacterial K^+^-ATPases, featuring a specialized nucleotide-binding pocket that ensures ligand specificity (Yu et al., 2018). Mutations like R778L disrupt intra-domain salt bridges in the P-domain, compromising phosphorylated state stability and attenuating ATP hydrolysis (Ayub et al., 2025; Xue et al., 2023). Cryo-electron microscopy further illustrates that ATP binding induces catalytic complex formation between N- and P-domains, driving transmembrane helix movements to reset the transporter after ion release (Yang et al., 2023; Concilli et al., 2020). This ATP-dependent cycle is fundamental to copper transport efficiency, directly impacting ATP7B functional integrity across cellular contexts (Li M. et al., 2021).

### 2.2 Key dynamic steps of the copper transport cycle

Although ATP7B shares the fundamental catalytic principles of P-type ATPases, its transport cycle is uniquely governed by an autoinhibitory mechanism mediated by its extended N-terminal tail—a feature absent in canonical P2-type pumps like SERCA (Yang et al., 2023; Bitter et al., 2022). Under physiological conditions, the cycle initiation requires not only substrate binding but also a specific structural release (Orädd et al., 2022). ATP7B receives Cu(I) from the cytosolic chaperone Atox1, a process involving tight interaction with the N-terminal MBD1–3 (Yu et al., 2017; Huang et al., 2014). Unique to ATP7B, this copper transfer acts as an allosteric switch: MD simulations and structural studies indicate that copper binding disrupts the inhibitory interface between MBD1–3 and the nucleotide-binding domain, inducing a transition towards a highly dynamic state that relieves the steric inhibition locking the catalytic core (Yu et al., 2018; Orädd et al., 2022).

Once “unlocked,” the transport cycle proceeds through distinct structural rearrangements. MBD6 serves as a docking unit at the transmembrane domain (TMD) entrance, bridging copper delivery into the high-affinity binding sites (Ponnandai Shanmugavel et al., 2017; Shanmugavel and Wittung-Stafshede, 2019). Upon copper entry, the enzyme transitions to the E1 state, where the metal is guided through a channel lined with thiol-containing residues in TM6 and TM7. This step is electrogenic and tightly coupled to the phosphorylation of the P-domain by ATP, forming the E1-P intermediate (Yang et al., 2023; Tadini-Buoninsegni, 2020).

The transition to the E2-P state is mechanically governed by the Actuator (A) domain. Structural analyses indicate that the A-domain undergoes a rotation that acts as a “structural lever,” physically transmitting the energy of ATP hydrolysis to the TMD (Yang et al., 2023; Bitter et al., 2022). This movement disrupts the MBD-TMD interface and triggers the rearrangement of transmembrane helices to close the cytosolic gate and open the luminal exit, releasing copper into the Golgi lumen (Yang et al., 2023). Finally, dephosphorylation resets the protein to the E2 state, and the subsequent closing of the luminal gate restores the E1 conformation for the next cycle. Dysfunction in any step of this tightly coupled, uniquely regulated structural choreography, whether in the N-terminal autoinhibition release or the A-domain lever mechanism, leads to the copper homeostasis imbalance observed in WD (Dev et al., 2022; Lutsenko et al., 2025).

### 2.3 Pathogenic mutations: structural disruption and functional consequences

ATP7B mutations cause WD, which can be classified into three types: missense mutations, truncating mutations, and splicing mutations (Concilli et al., 2020; Zhou et al., 2025; Nayagam et al., 2023). Missense mutations, the most prevalent class, typically involve single amino acid substitutions that compromise protein stability or catalytic efficiency without abolishing synthesis. Notable examples include H1069Q in the N-domain and R778L in the TMD, which are frequently associated with specific ethnic distributions and phenotypic presentations (Yang et al., 2023; Xue et al., 2023; Lao and Le, 2024).

Truncating mutations typically introduce premature stop codons, leading to incomplete ATP7B synthesis and the loss of critical domains such as the P-domain or TMD region, which severely disrupts copper transport (Zhang et al., 2022; Chaudhuri et al., 2022). For example, the c.813C>A (p.C271X) mutation truncates the P-domain, abolishing ATP hydrolysis capacity and impairing copper translocation (Chaudhuri et al., 2022; Gul et al., 2022). Additionally, splicing mutations affect RNA splicing sites, causing transcript misprocessing or exon skipping, such as the c.1543 + 1G>C mutation triggers exon 3 skipping, leading to ER retention of mutant ATP7B (Zhou et al., 2025). Table 1 summarizes the functional impact of various representative mutations on different ATP7B domains.

**TABLE 1:** Molecular classification and functional impacts of ATP7B pathogenic mutations.

Mutation type	Representative mutation	Affected domain	Functional impact	Literature support
Missense mutation	H1069Q	N-domain	Disrupts ATP binding site, leading to ER retention and loss of copper transport function	Concilli et al. (2020)
Missense mutation	R778L	TMD	Alters transmembrane channel structure, impairing copper transport function	Li et al. (2021b); Xue et al (2023)
Truncating mutation	c.813C>A (p.C271X)	P-domain	Truncates P-domain, abolishing ATP hydrolysis and copper translocation	Chaudhuri et al (2022); Gul et al. (2022)
Splicing mutation	c.1543 + 1G>C	Splice site (exon 3)	Triggers exon 3 skipping, leading to ER retention and proteasomal degradation	Zhou et al. (2025)

## 3 MD simulation techniques: decoding mechanisms at atomic scale

### 3.1 Principles of MD simulation and innovations in ATP7B simulations

MD simulation is a computational technique that solves Newton’s equations of motion to follow the time‐dependent positions of all atoms in a molecular system. Unlike static structural methods (e.g., X‐ray crystallography or cryo‐EM), which provide only single conformational snapshots, MD yields continuous trajectories and can capture dynamic conformational transitions and allosteric changes at atomic resolution (Wang et al., 2024; Grewal et al., 2025; Wu et al., 2022). For example, the first cryo‐EM structure of ATP7B provided a static “snapshot” of the transporter in one state (Bitter et al., 2022). In contrast, MD studies reveal the protein’s intrinsic motions. Efremov et al. built a model of the ATP‐binding (N) domain of ATP7B based on the SERCA pump and found that long, all-atom MD trajectories produced large-scale domain motions and “closure‐type” transitions that would be invisible in static structures (Efremov et al., 2004). Similarly, extensive MD of the six metal‐binding domains (MBDs) showed domain‐specific flexibility: loops in MBD4–MBD5 were unusually mobile, whereas other domains were more rigid (Rodriguez-Granillo et al., 2009). Interdomain MD simulations further revealed correlated, cooperative motions among connected domains (Rodriguez-Granillo et al., 2010), consistent with long-range allosteric communication. MD thus provides atomic‐scale insight into conformational plasticity and allostery in ATP7B that complements static structural data (Rodriguez-Granillo et al., 2010; Hercend et al., 2011).

Before high-resolution structures of full-length ATP7B were available, researchers have relied on modeling to fill the gaps. Homology modeling using related P‐type ATPase structures (especially the Ca2+‐ATPase SERCA) has been a key strategy. For instance, Efremov et al. generated a 3D model of the ATP7B N‐domain by aligning it to SERCA’s E1/E2 conformations (Efremov et al., 2004), then performed MD to refine the model. Such homology‐based models have guided understanding of ligand‐binding sites and disease mutations. In parallel, solution structures of individual domains have been obtained experimentally: Yu et al. solved the NMR structure of ATP7B’s first metal‐binding domain (MBD1), the last unsolved MBD, revealing how a Wilson‐disease mutation (G85V) disrupts its fold (Yu et al., 2018). More recently, AI‐driven prediction tools have been applied. Stalke et al. used an AlphaFold model of full‐length ATP7B to interpret the effects of patient variants, finding that structural modeling on the AF2 predicted structure allowed reclassification of several variants as (likely) pathogenic (Stalke et al., 2023). In summary, a combination of homology modeling (from SERCA and other P‐ATPases), experimental domain structures (e.g., NMR/SAXS of MBDs), and AlphaFold predictions has been used to build working models of ATP7B domains and full‐length protein in the absence of complete experimental structures (Stalke et al., 2023; Yu et al., 2018; Efremov et al., 2004). These structural models provide critical initial conformations for MD simulations, and their integration with dynamic simulations can further reveal the mechanisms underlying the impact of mutations on ATP7B function.

Furthermore, MD simulations have evolved to rigorously account for the lipid membrane environment to capture the physiological dynamics of the TMD. Whether utilizing explicit phospholipid bilayers (Orädd et al., 2022) to introduce realistic lateral pressure, or employing implicit membrane models (Braiterman et al., 2014) to enforce hydrophobic constraints, establishing this energetic framework is vital for modeling transport cycles and identifying pathogenic instabilities (Cymer et al., 2015; Phillips et al., 2009). For instance, the S653Y mutation involves a bulky tyrosine side chain that creates a steric clash between TM1 and TM2 (Braiterman et al., 2014). It is the energetic constraint imposed by the membrane environment that reveals how this packing defect forces a helical displacement, thereby explaining the mutation-induced ER retention. Thus, these findings suggest that by accurately representing the membrane’s physical restrictions, computational modeling can provide a robust basis for distinguishing between benign polymorphisms and pathogenic packing defects.

A critical challenge in interpreting MD simulations lies in the “timescale gap’” be-tween simulated trajectories (typically nanoseconds to microseconds) and real biological functions (milliseconds to seconds) (Wang et al., 2024). While standard MD cannot directly reproduce the kinetics of the entire copper transport cycle, the studies reviewed herein bridge this gap by focusing on local conformational thermodynamics rather than absolute kinetics. Specifically, simulations capture fast “precursor” events—such as the destabilization of local hy-drogen bond networks or increased loop flexibility (as seen in H1069Q and G85V mu-tants)—which serve as early mechanistic triggers for slower, macroscopic functional de-fects (Efremov et al., 2004; Kumar et al., 2017). Furthermore, the biological relevance of these “arbitrary” simulation time-scales is validated through comparative analysis: the relative divergence in dynamic be-havior between wild-type and mutant structures provides a robust proxy for predicting functional impairment, which is consistently corroborated by experimental validation (e.g., thermal stability assays and transport measurements) (Yu et al., 2018; Wu et al., 2022).

The following sections detail how MD simulations have been employed to investigate mutational effect mechanisms, copper binding and transport pathways, protein-protein interactions, and drug binding simulations.

### 3.2 Mutational effect mechanisms

The H1069Q mutation, the most prevalent Wilson disease (WD)-causing variant in the N-domain, disrupts ATP binding and hydrolysis (Shanmugavel et al., 2019; Calvo et al., 2025). Building on this, Efremov et al. independently constructed homology models of the E1/E2 states. Through μs-scale molecular dynamics (MD) simulations in explicit solvent, they observed that H1069Q impairs the “closure-type” motions essential for bringing phosphate and adenosine moieties into proximity, correlating with a 2-fold reduction in ATP affinity (Efremov et al., 2004). Complementary studies by Rodrigwald et al. on the isolated N-domain carrying H1069Q revealed that substituting His1069 with Gln disrupts the local hydrogen-bond network surrounding the ATP-binding pocket (Rodriguez-Granillo et al., 2008). This disruption increases loop flexibility compared to wild-type, compromising domain closure necessary for catalysis (Rodriguez-Granillo et al., 2008). Further elucidating the mechanism, Hercend et al. combined molecular modeling and surface plasmon resonance to show that H1069Q disrupts the Mg^2+^-ATP coordination site via conformational changes in a flexible loop of the N-domain, impairing nucleotide binding (Hercend et al., 2011). Beyond these local structural changes, Berghe et al. demonstrated that H1069Q also reduces ATP7B protein expression and causes ER retention, indicative of protein misfolding. Significantly, this defect was partially rescued by pharmacological chaperones, providing strong support for a folding deficiency underlying the mutation (van den Berghe et al., 2009). Collectively, these studies converge to show that H1069Q induces a hierarchical failure: local destabilization of the ATP-binding pocket propagates to impair the global “closure-type” domain dynamics, thereby arresting the transport cycle at the catalytic phosphorylation step.

Mutations within the metal-binding domains (MBDs) of ATP7B impair copper transfer and allosteric regulation (Kumar et al., 2017; Ovchinnikova et al., 2024). Specifically, MD simulations demonstrated that G85V (MBD1) and G591D (MBD6) act primarily by destabilizing the domain structure and increasing its conformational dynamics, rather than directly disrupting the copper-binding site, which provided atomistic insights into the dynamic perturbations and structural changes underlying the experimentally observed thermal destabilization and misfolding (Kumar et al., 2017). Further elucidating the mechanism of G85V, Yu et al. showed that this mutation disrupts a conserved glycine hinge in MBD1, reducing thermal stability and impairing both copper binding and chaperone (Atox1)-mediated transfer. Critically, their MD trajectories indicated that G85V induces long-range conformational changes that disrupt interdomain interactions essential for ATP7B activation, demonstrating how local amino acid substitutions in distant loops compromise global copper transport via allosteric effects (Yu et al., 2018). Together, these findings highlight that disease-associated MBD mutations act by severing the long-range communication between the regulatory N-terminus and the catalytic core, effectively locking the transporter in an auto-inhibited state and preventing cycle initiation.

Building on this concept of long-range effects, Shanmugavel et al. employed 1.5-μs MD simulations to demonstrate that mutations in MBD5/6 remotely impact the dynamics of the copper-binding loop (CXXC motif) (Shanmugavel et al., 2019). Specifically, L492S increased the overall dynamics of MBD5, R616W significantly enhanced fluctuations specifically within the copper-binding loop, and A604P induced moderate dynamic changes in the loop while reducing α-helical content. Notably, G626A conferred greater structural rigidity to the domain (Shanmugavel et al., 2019). These results suggest that Wilson disease mutations in ATP7B MBDs primarily disrupt function via allosteric destabilization of the copper-binding loop, with MD simulations effectively capturing these long-range perturbations. However, the exceptional rigidity induced by G626A highlights the existence of alternative pathogenic mechanisms—such as impaired regulatory interactions—beyond direct copper transport defects, underscoring the complexity of genotype-phenotype correlations in WD (Shanmugavel et al., 2019).

The A domain plays a critical role in conformational transitions during the ATPase cycle. However, missense mutations in transmembrane domains (TMs), such as S653Y, disrupt copper export by altering helix packing and dynamics (Huster et al., 2012). Specifically, the S653Y mutation in TM1 causes local distortion in TM1--TM2 interactions due to the bulky tyrosine substitution. This effect was validated by molecular dynamics (MD) simulations, which demonstrated TM1 displacement and disrupted helical packing (Braiterman et al., 2014). Consequently, this helical misalignment abolishes copper-responsive trafficking from the trans-Golgi network, despite preserved transport activity (Braiterman et al., 2014). Thus, S653Y mutations exemplify how local structural perturbations in helix packing translate into a global failure of the trafficking cycle, preventing the transporter from reaching its functional location at the canalicular membrane without abolishing intrinsic catalytic activity.

**FIGURE 2:**
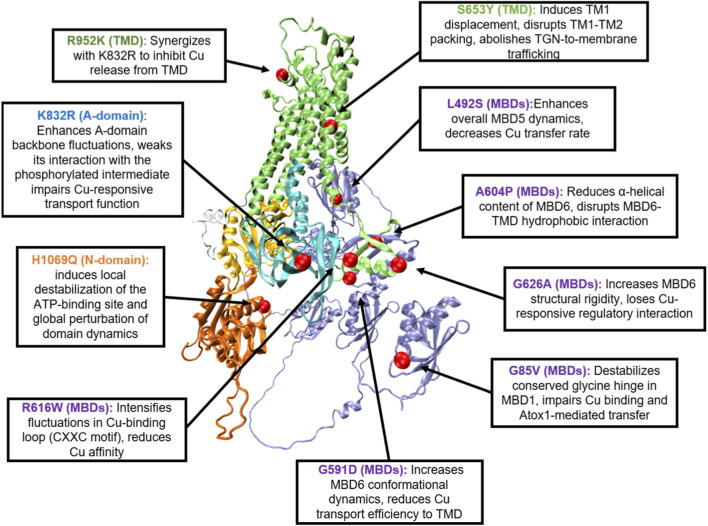
Molecular Dynamics-Probed Pathogenic Mutations on the ATP7B Protein Structure. The three-dimensional structure of human ATP7B is based on the AlphaFold v3.0 prediction. Structural domains are color-coded as follows: MBDs in light purple, TMD in light green, N-domain in light orange, P-domain in light yellow, A-domain in light blue, and C-terminal region in light gray. Pathogenic mutation sites that have been extensively characterized through molecular dynamics (MD) simulations are highlighted as red spheres, including G85V (MBD1), G591D (MBD6), L492S (MBD5), R616W (MBD6), A604P (MBD6), G626A (MBD6), S653Y (TMD), H1069Q (N-domain), K832R (A-domain), and R952K (TMD). The text in the box shows how they disrupt copper transport through diverse mechanisms, including destabilization of functional loops, impairment of allosteric regulation, and disruption of domain-domain interactions.

Single nucleotide polymorphisms (SNPs) represent significant genetic modifiers that fine-tune copper homeostasis through structural-dynamic mechanisms. Two common SNPs in *ATP7B* (K832R and R952K) alter protein conformational dynamics, impairing ATP7B stability, intracellular localization, and copper transport function to modulate systemic copper balance (McCann et al., 2019). Specifically, the K832R variant exhibits elevated backbone fluctuations, an expanded radius of gyration, and increased solvent accessibility (SASA) at residues near the mutation site and the distal TGE motif (T858, E860) (McCann et al., 2019). These local perturbations compromise both the global dynamic stability and allosteric regulatory capacity of the A-domain, weakening its interaction with the phosphorylated intermediate and thereby reducing the efficiency of the conformational transitions required for copper release. In contrast, R952 localizes to the luminal loop between TM3–TM4; MD simulations revealed no significant dynamic alterations for this residue alone. Notably, however, R952K synergizes with K832R to suppress copper transport (McCann et al., 2019). Mechanistically, K832R-induced A-domain flexibility likely cooperates allosterically with R952K to inhibit copper release—paralleling functions of the homologous loop in ATP7A. Collectively, these findings demonstrate that SNPs modulate copper homeostasis via subtle yet cumulative dynamic perturbations, acting as phenotypic modifiers in neurodegenerative disorders (e.g., Alzheimer’s disease) and Wilson disease, thus offering novel targets for precision medicine. Table 2 serves as a summary and visual consolidation of the detailed molecular dynamics (MD) findings for various ATP7B mutations (e.g., G85V, H1069Q, K832R) that are discussed throughout Section 3.2. Figure 2 directly illustrates the mutations listed in Table 2.

**TABLE 2:** Molecular dynamics insights into ATP7B pathogenic mutations.

Mutation	Structural domain	MD simulation findings	Functional consequence	Literature support
G85V	MBDs	Disrupts a conserved glycine hinge; induces long-range conformational changes that disrupt interdomain interactions	Reduces thermal stability; impairs both copper binding and chaperone (Atox1)-mediated transfer	Yu et al. (2018); Kumar et al. (2017)
G591D	MBDs	Destabilizes the domain structure and increases its conformational dynamics	Reduces copper transport efficiency	Kumar et al. (2017)
L492S	MBDs	Markedly enhances the overall structural dynamics of MBD5; enhances fluctuations in the copper-binding loop	Causes ER retention and protein mis-localization; reduces the copper transfer rate from the chaperone Atox1	Shanmugavel et al. (2019)
R616W	MBDs	Significantly enhances fluctuations specifically within the copper-binding loop (CXXC motif)	Reduces copper affinity and impairs function	Shanmugavel et al. (2019)
A604P	MBDs	Induces dynamic changes in the Cu-loop; reduces α-helical content	Disrupts MBD6-TMD hydrophobic interaction and impairs function	Shanmugavel et al. (2019)
G626A	MBDs	Confers greater structural rigidity; increases secondary structure content	Loses copper-responsive regulatory interactions	Shanmugavel et al. (2019)
S653Y	TMD	Causes TM1 displacement and disrupts TM1-TM2 helical packing	Abolishes copper-responsive trafficking from the trans-Golgi network	Braiterman et al., 2014
H1069Q	N-domain	Disrupts the local H-bond network; increases loop flexibility; impairs “closure-type” motions; perturbs Mg^2+^-ATP coordination	Reduces ATP binding affinity; causes protein misfolding and ER retention	Efremov et al. (2004); Hercend et al. (2011); Rodriguez-Granillo et al. (2008); van den Berghe et al. (2009)
K832R	A-domain	Elevates backbone fluctuations and increases solvent accessibility; increases conformational flexibility	Weakens interaction with the phosphorylated intermediate, diminishing copper-responsive transport function	McCann et al (2019)
R952K	TMD	No significant dynamic alterations for this residue alone (simulations show synergy with K832R)	Synergizes with K832R to suppress copper transport; acts as a phenotypic modifier	McCann et al. (2019)

### 3.3 Copper binding and transport pathways

MD simulations have emerged as a pivotal tool for elucidating the dynamic characteristics of copper-binding sites in ATP7B. Rodriguez-Granillo et al. employed MD simulations to analyze the conformational dynamics of individual MBDs of ATP7B in both apo (copper-free) and holo (copper-bound) states (Rodriguez-Granillo et al., 2009). Their findings revealed that copper binding significantly reduced the structural flexibility of all MBDs (except MBD4) and uncovered distinct dynamic variations among different MBDs. Through MD simulations of the full-length ATP7B protein, Orädd et al. provided the first atomic-level insights into its copper-dependent allosteric mechanism (Orädd et al., 2022). Following copper binding, the overall dynamics of the N-terminal regulatory region of ATP7B markedly increased (Orädd and Andersson, 2022). Specifically, MBD2 and MBD3 exhibited the highest mobility upon copper binding, leading to an extended conformation of the N-terminal tail and thereby relieving auto-inhibition at the interface of the core domains (A/N domains) (Orädd et al., 2022). Concurrently, MBD5 moved closer to the copper entry site within the transmembrane domain (M-domain), potentially facilitating the preparation for copper transfer into the transport pathway (Orädd et al., 2022). This dynamic coupling effect is corroborated by Yu et al. in their study of MBD1: The conformational flexibility of Gly^85^ in MBD1 confers high mobility to the β2-β3 loop, which is crucial for maintaining the geometric stability of the copper-binding site (Yu et al., 2018). Alterations in the side chain volume and hydrophobicity of this residue can rigidify the β2-β3 loop conformation, disrupt the local hydrogen-bonding network, and consequently perturb the microenvironment of the copper-binding site. The cysteine motifs exhibit high affinity for Cu(I) binding (Jung et al., 2020). Cys-to-Ser mutations are frequently employed in *vitro* studies to compare functional proteins with non-functional/inhibited counterparts (Jung et al., 2020; Philips et al., 2015; Pavlin et al., 2019). MD simulations have demonstrated that while Cys→Ser mutants retain Cu(I)-binding capability, they lead to functional impairment by altering local dynamics, hindering proton-coupled metal transfer, and promoting electrostatically mediated aberrant aggregation (Pavlin et al., 2019). These findings provide atomistic insights into copper homeostasis regulation mechanisms (Pavlin et al., 2019).

MD simulations have revealed a stepwise cooperative mechanism for copper transfer from the cytosolic chaperone Atox1 to ATP7B. Rodriguez-Granillo et al. employed MM-PBSA calculations to determine the binding energies between Atox1 and individual MBDs, identifying the Atox1-WD4 complex as exhibiting the strongest interaction (Rodriguez-Granillo et al., 2009). This preferential binding arises from the high conformational flexibility of MBD4 in its apo state, facilitating the formation of transient binding intermediates with Atox1 (Rodriguez-Granillo et al., 2009). This finding provides a molecular explanation for the experimentally observed preferential copper transfer to MBD4, MBD5, and MBD6. Using combined quantum mechanics/molecular mechanics (QM/MM) simulations, the authors further postulated a three-stage copper transfer mechanism from Atox1 to MBD4: (i) initial formation of a three-coordinate intermediate involving Cys^370^ of MBD4 and Cu(I) bound to Atox1; (ii) subsequent coordination by Cys^373^ of MBD4 accompanied by rotation of the α1 helix; and (iii) final dissociation of Cys^32^ from Atox1, resulting in complete Cu(I) transfer to the Cys^370^-Cys^373^ site within MBD4 (Rodriguez-Granillo et al., 2009). Further studies demonstrated that copper delivery by Atox1 to the MBD1-3 cluster significantly reduces interdomain interactions within this group (Yu et al., 2017). This dynamic decoupling relieves the autoinhibitory effect exerted by MBD1-3 on the catalytic core of ATP7B, thereby activating its ATP hydrolysis activity (Yu et al., 2017). The critical role of domain cooperativity was further elucidated by Shanmugavel et al. The L492S mutation in MBD5 markedly enhanced its overall structural dynamics, consequently reducing the copper transfer rate (Shanmugavel et al., 2019). This observation underscores the decisive influence of structural rigidity on the efficiency of the transfer pathway. Importantly, this interdomain cooperativity is modulated by the intracellular environment. MD simulations demonstrated that physiologically relevant variations in pH and salt concentration significantly alter the relative motion and fluctuation between the MBD5 and MBD6 domains by perturbing the surface charge network of the MBD5/6 construct. High-fluctuation states (low pH/high salt) promote domain decoupling, reduce thermal stability, and impair copper transfer efficiency from the chaperone Atox1. Conversely, low-fluctuation states (high pH/low salt) enhance domain cooperativity and functional activity, elucidating the regulatory mechanism governing ATP7B function within the dynamic intracellular milieu (Nilsson et al., 2013).

### 3.4 ATP binding mechanism

MD simulations have provided atomistic insights into the ATP-binding mechanism and functional regulation of ATP7B. Early studies employing homology modeling and MD simulations first revealed the three-dimensional conformational characteristics of the N-domain (Efremov et al., 2004). Analysis of the root mean square fluctuation (RMSF) values indicated significantly higher conformational flexibility in the N-domain compared to the P-domain (Efremov et al., 2004). This inherent flexibility is postulated to be functionally relevant for ATP binding. Molecular docking studies validated a dual-site ATP binding mechanism: adenosine embeds within a hydrophobic cleft formed by residues H1069, R1151, and D1164, while the phosphate moiety anchors via hydrogen bonding near the catalytic residues D1027, K1028, and T1029 (Rodriguez-Granillo et al., 2008). The simulations further suggested that large-scale conformational motions of the domain may facilitate the cooperative engagement of both binding sites (Rodriguez-Granillo et al., 2008). This structural framework established the basis for mutational analysis.

Further studies revealed that allosteric regulation of the N-domain relies on flexible structural elements. Hercend et al. combined MD simulations with experimental validation to demonstrate that Mg^2+^-ATP binding induces a conformational change in a long flexible loop within the N-domain (Hercend et al., 2011). This loop regulates ATP binding and dissociation through steric hindrance, uncovering the dynamic nature of an allosteric switch (Hercend et al., 2011). Furthermore, contrasting with the proposal by Rodriguez-Granillo et al. that H1069 fine-tunes ATP orientation, their work confirmed that H1069 does not directly participate in ATP binding. Instead, this residue likely influences phosphorylation by mediating interactions between the N- and P-domains (Rodriguez-Granillo et al., 2008). Similarly, MD simulations of SNP mutations (e.g., K832R) indicated increased surface residue exposure and elevated conformational flexibility within the actuator domain (A-domain) (McCann et al., 2019). These alterations weaken its interaction with the phosphorylated intermediate, leading to diminished copper-responsive transport function, which underscores the indirect regulatory role of non-catalytic domains on ATP hydrolysis.

The core regulation of ATP-binding activity stems from copper-induced global conformational rearrangements. Yu et al. utilizing molecular docking-assisted SAXS and NMR analyses, discovered that copper delivery by the chaperone Atox1 to the MBD1-3 cluster significantly enhances the dynamic freedom of this region (Yu et al., 2017). This promotes a transition of MBD1-3 from a closed to an open conformation. This conformational change physically displaces the N-terminal peptide from its inhibitory binding site on the N-domain, thereby activating ATPase activity (Yu et al., 2017). This mechanism is extended by MD simulations of full-length ATP7B: Copper binding induces increased dynamics in MBD2, MBD3, and MBD5 (Orädd et al., 2022),which promotes dissociation of the MBD1-3 cluster from the core catalytic domains (A/P/N) while simultaneously positioning MBD5 closer to the transmembrane copper channel (Orädd et al., 2022). These concerted changes are thus proposed to synergistically facilitate the coupling of ATP hydrolysis to copper transport, providing a structural hypothesis for the experimentally observed functional coupling.

## 4 Data-driven phenotype prediction and mechanistic insights

### 4.1 Epigenetic and transcriptional regulatory networks

While molecular dynamics elucidate the atomic origins of ATP7B dysfunction, translating these micro-scale structural insights into macroscopic clinical utility requires scalable, data-driven approaches. Consequently, the phenotypic heterogeneity of WD stems not only from ATP7B gene mutations but is also profoundly influenced by the epigenetic regulatory network. Computational biology techniques have emerged as pivotal tools for deciphering this complexity. Machine learning analysis of whole-genome methylation profiles, for instance, has unveiled WD-specific epigenetic signatures. Mordaunt et al. (2019) employed whole-genome bisulfite sequencing (WGBS) combined with clustering algorithms to identify 1,840 differentially methylated regions (DMRs) in the livers of WD patients (Mordaunt et al., 2019). Notably, hypermethylated DMRs were significantly enriched at binding sites for hepatic developmental transcription factors, including HNF4A and FOXA1 (Mordaunt et al., 2019). Further analysis of blood-derived DMRs using a random forest model enabled constructing a classifier capable of distinguishing hepatic from neurologic WD subtypes. This classifier, based on 44 core DMRs and achieving a predictive performance of AUC 0.9, provides a potential algorithmic foundation for non-invasive phenotypic subtyping (Mordaunt et al., 2019).

At the mechanistic level, multi-omics integrative analysis facilitates dynamic modeling of epigenetic-transcriptional regulatory networks. Sarode et al. integrated RNA-seq and ChIP-seq data from tx-j mice (Sarode et al., 2021). Differential expression and pathway enrichment analyses revealed that copper accumulation suppresses HDAC4/5 expression, consequently elevating histone H3K9ac and H3K27ac modifications and activating causal pathways involving lipid metabolism-related genes such as PPARγ. This delineates a logical framework for selecting intervention targets (Sarode et al., 2021). Complementing this, Höflich et al. utilized the ConSeq tool to quantify sequence conservation within the ATP7B core promoter region across 91 mammalian species (Höflich et al., 2021). Coupled with ElemeNT predictions of initiator (Inr) elements, they precisely mapped transcription start sites (TSSs) clustered near the translation start site (Höflich et al., 2021). Multi-species sequence alignment demonstrated that rare variants in this core promoter region significantly impair transcriptional activity (Höflich et al., 2021). While common polymorphisms show no direct impact on transcription efficiency, they may contribute to phenotypic heterogeneity through genetic modifier effects, offering an explanation at the genetic modification level.

Computational models further elucidate dynamic environment-epigenetic interactions. Medici et al. analyzed the embryonic liver transcriptome of tx-j mice via RNA-seq, identifying dysregulated genes in pathways like oxidative phosphorylation (Medici et al., 2016). Principal component analysis (PCA) of expression patterns indicated that maternal choline supplementation partially restored transcriptional levels of these genes, providing quantitative support for nutrient intervention in modulating the WD-associated transcriptome (Medici et al., 2016). These epigenetic perturbations further disrupt mitochondrial metabolism (e.g., TCA cycle), as quantified by plasma metabolomics in Medici’s ML model. Collectively, these computational approaches significantly expand our understanding of the WD regulatory network and provide system-level insights into its phenotypic heterogeneity.

### 4.2 Machine learning-based phenotype prediction

WD, a disorder of copper metabolism caused by mutations in the *ATP7B* gene, exhibits pronounced heterogeneity in both its clinical manifestations and genetic background (Alonso-Castellano et al., 2025; Benzine et al., 2025). Clinical presentations are highly heterogeneous (hepatic, neurological, or mixed forms), and the genotype-phenotype correlations are complex (Petruzzelli et al., 2025; Wang et al., 2025b). In recent years, machine learning (ML) and artificial intelligence (AI) techniques have emerged as powerful tools to enhance mutation pathogenicity prediction, phenotypic classification, and early diagnosis through integration of multi-dimensional data (genetic, imaging, clinical indicators) (Bajwa et al., 2021; Wang et al., 2025b; Vo et al., 2024; Stremmel et al., 2025; Liang et al., 2023). To provide a structured overview of these diverse methodologies, we summarize the key algorithms, input features, and clinical applications of representative studies in Table 3.

**TABLE 3:** Summary of ML applications in WD prediction and diagnosis.

Application domain	Methodology/Algorithm	Input data/Features	Key outcome/Performance	Literature support
Mutation pathogenicity	WilsonGenAI (TabNet and XGBoost)	ATP7B variant annotations (ACMG/AMP criteria)	Automated classification of pathogenic vs. benign variants; achieved 91.4% concordance between models	Vatsyayan et al. (2024)
Liver cirrhosis prognosis	XGBoost	Routine clinical data (e.g., P-LCC, RDW-CV, MCV)	Non-invasive prediction of liver cirrhosis risk based on hematological parameters	Chen et al. (2022)
Neurological symptom prediction	XGBoost	Clinical indicators (e.g., brainstem injury, serum creatinine, age)	Identified top predictors for early intervention in neurological WD; first model for this specific phenotype	Yang et al. (2024)
Phenotypic subtyping	Artificial neural networks (ANN)	Plasma amino acid levels (glutamate, asparagine, taurine)	Distinguished hepatic, neurological, and asymptomatic subtypes; 100% accuracy in distinguishing WD from healthy controls	Medici et al. (2016)
Neuroimaging diagnosis	Optimized 3D deep CNN (iDCNN)	Brain MRI datasets	Achieved 98.28% accuracy in tissue classification despite limited sample size; identified white matter hyperintensity patterns	Agarwal et al (2021)
Neurological deterioration risk	Explainable XGBoost	MRI radiomics (T1WI) and clinical features (UWDRS-N score)	Predicted neurological deterioration during anti-copper therapy; utilized SHAP analysis to prioritize risk factors	Wang et al. (2025c)

In mutation pathogenicity classification, Vatsyayan et al. (2024) developed the WilsonGenAI model (based on TabNet and XGBoost) (Vatsyayan et al., 2024). Trained on an ACMG/AMP-annotated dataset of *ATP7B* variants, this model automates the classification of pathogenic versus benign mutations, enabling efficient screening of variants of uncertain significance (VUS) (Vatsyayan et al., 2024). Notably, the two constituent models achieved concordant classifications for 91.4% of analyzed variants (726 pathogenic +167 benign), significantly improving clinical interpretation efficiency (Vatsyayan et al., 2024).

Beyond variant annotation, ML offers novel insights into genotype-phenotype associations (Shribman et al., 2022). For example, Chaudhuri et al. identified the hotspot mutation c.813C>A in an Eastern Indian WD cohort but observed no significant association with key clinical phenotypes (e.g., cognitive scores, imaging features) (Chaudhuri et al., 2022). This weak correlation was further corroborated by ML models (logistic regression and random forest), which indicated that gait abnormalities, age at diagnosis, dystonia, and depression were critical predictors for common mutations (Chaudhuri et al., 2022).

In prognosis prediction, Chen et al. pioneered a non-invasive XGBoost model to predict liver cirrhosis in WD using routine clinical data, hematological parameters, urinary copper, and serum ceruloplasmin (Chen et al., 2022; Martínez-Morillo and Bauça, 2022). Their analysis revealed platelet-large cell count (P-LCC), red blood cell distribution width coefficient of variation (RDW-CV), and mean corpuscular volume (MCV) as key biomarkers, providing a data-driven tool for clinical risk stratification (Chen et al., 2022). Similarly, Yang et al. (2024) developed the first XGBoost model to predict neurological symptoms, identifying brainstem injury, elevated serum creatinine, advanced age, high indirect bilirubin, and low ceruloplasmin as top predictors for early intervention (Yang et al., 2024). Furthermore, artificial neural networks (ANN) exhibit unique value in the refined phenotypic subtyping of WD. Medici et al. developed a diagnostic model capable of distinguishing between the hepatic type (WDH), neurological type (WDN), and asymptomatic type (WDA) of WD by integrating plasma amino acid levels (glutamate, asparagine, taurine) and the Fischer ratio (Wang Y. et al., 2025). This model distinguishes WD patients from healthy individuals with 100% accuracy and achieves a predictive confidence of 69% for the WDN subtype. Mechanistically, these metabolic markers are closely associated with disturbances in the urea cycle and tricarboxylic acid cycle, confirming that mitochondrial dysfunction constitutes the core pathological basis driving phenotypic differences, which provide a metabolomics-driven machine learning approach for non-invasive subtyping of WD (Wang Y. et al., 2025).

Innovations in multimodal diagnostics have also advanced WD detection (Zhan et al., 2024). In neuroimaging, Agarwal et al. created an optimized 3D deep convolutional neural network (iDCNN) for WD brain MRI analysis, achieving 98.28% accuracy despite limited training data (*n* = 46) (Agarwal et al., 2021). Through feature map intensity and bispectral validation, the model confirmed distinctive white matter hyperintensity patterns, establishing a new AI benchmark for WD radiological diagnosis (Agarwal et al., 2021). Complementing this, Wang et al. built an explainable XGBoost model integrating T1WI radiomics and clinical features to predict neurological deterioration during anti-copper therapy (Wang et al., 2025c). Leveraging SHAP analysis, their model prioritized UWDRS-N score, age, and specific texture features (e.g., putamen GrayLevelNonUniformity) as key predictors for clinical risk stratification (Wang et al., 2025c). Additionally, the application of machine learning in deciphering the mechanisms of copper metabolism-related diseases has extended to the cardiovascular field. Based on the cuproptosis pathway, Tan et al. employed algorithms such as SVM and XGBoost to integrate transcriptomic and clinical data, identifying core genes including ATP7B and DLST as diagnostic biomarkers for ischemic cardiomyopathy (IC). Their constructed SVM model demonstrated exceptional performance (AUC = 0.914) (Tan et al., 2024). Notably, ATP7B—a key pathogenic gene in Wilson disease—was further validated in this study to participate in cuproptosis regulation in IC, highlighting its pivotal role in cross-disease copper metabolism networks.

Critically, the reliability and clinical applicability of these machine learning models depend on rigorous data management to prevent overfitting and bias arising from subjective data splitting. To address this, recent high-quality studies have adopted diverse validation strategies tailored to their specific dataset characteristics. For variant pathogenicity classification, Vatsyayan et al. went beyond internal splitting by utilizing a completely independent validation dataset of ACMG-classified variants, ensuring that the model’s performance was robust to unseen genetic data (Vatsyayan et al., 2024). In the context of clinical prognosis, Chen et al. employed a strictly separated held-out test set (30% of the cohort) to evaluate their XGBoost model. The performance gap observed between training (AUC 0.99) and testing (AUC 0.79) in their study highlights the vital importance of such split-sample validation in detecting potential overfitting and establishing realistic performance expectations (Chen et al., 2022). Furthermore, addressing the challenge of limited data in rare diseases, Agarwal et al. implemented data augmentation techniques alongside bispectral analysis to validate that the model’s learned features corresponded to true pathological signals rather than artifacts of a specific random split (Agarwal et al., 2021). Moving forward, the adoption of nested cross-validation (Wang et al., 2025c) has been suggested as a standard for studies involving complex model selection, as it offers the highest protection against data leakage (Vabalas et al., 2019). This framework, which strictly separates hyperparameter tuning from performance evaluation, offers the highest protection against data leakage and is essential for developing trustworthy AI tools for Wilson disease.

Beyond clinical and omics data, molecular dynamics (MD) simulations provide a rich source of atomic-level features for ML model development. (Liu et al., 2018; Wu et al., 2022). However, the raw output of MD—time-evolving atomic coordinates—is not directly compatible with most ML algorithms due to its high dimensionality, temporal correlation, and lack of fixed representation (Wang et al., 2024). Therefore, a critical step is feature extraction, where biophysically meaningful descriptors are computed from MD trajectories. These can include metrics of local flexibility (e.g., root-mean-square fluctuation), stability of secondary structure elements, persistence of key hydrogen bonds or hydrophobic contacts, and distances between functional sites (Efremov et al., 2004; Rodriguez-Granillo et al., 2008; Pavlin et al., 2019). Such engineered features transform dynamic structural information into a fixed-length numerical vector that can be integrated with genomic or clinical data to train classifiers for variant pathogenicity or predictors of phenotypic severity (Vatsyayan et al., 2024; Wu et al., 2023). Alternatively, emerging deep learning architectures, such as graph neural networks, can operate directly on graph-based representations of protein structures, learning relevant features end-to-end (Zhu et al., 2025). This integrative approach holds the promise of building more accurate and mechanistically interpretable models by grounding ML predictions in physical dynamics.

## 5 Computational integration for precision medicine: multi-omics subtyping and therapeutic targeting

### 5.1 Multi-omics network analysis for disease subtyping

Beyond the phenotypic predictions achieved by machine learning, understanding the systemic biological context of these predictions necessitates a multi-omics perspective to unveil therapeutic targets. In recent years, the integration of multi-omics technologies with computational biology has significantly advanced the exploration of Wilson’s disease (WD) mechanisms and target discovery (Zhang et al., 2021). Specifically, the consolidation of transcriptomic, proteomic, methylomic, and other omics data, coupled with computational approaches to construct systemic regulatory networks, provides a comprehensive perspective for unraveling WD’s complex pathological mechanisms (Wang et al., 2023).

A pivotal method for integrating these multi-omics data is co-expression network analysis, which enables the identification of key gene modules closely associated with disease phenotypes. For example, Zhang et al. established an lncRNA-mRNA network using coding-noncoding co-expression (CNC) analysis, revealing critical lncRNAs (e.g., Meg3, H19) that significantly correlate with liver fibrosis-related pathways (e.g., PPAR, MAPK signaling) and likely contribute to WD liver injury by directly regulating target genes (Zhang et al., 2021). Wang et al. further analyzed the full transcriptome of hippocampal tissues in tx-J mice, constructing ceRNA networks (circRNA-miRNA-mRNA and lncRNA-miRNA-mRNA) to screen key genes (e.g., Fosb, Shank3) linked to cognitive impairment (Wang et al., 2023). Notably, these genes were enriched in biological functions such as calcium signaling and cellular processes, offering novel insights into the molecular mechanisms of cognitive deficits in WD (Wang et al., 2023). Further demonstrating the power of this approach, Panzade et al. integrated mRNA-seq, miRNA-seq, and whole-genome bisulfite sequencing (WGBS) data, demonstrating that the Atp7b gene is co-regulated by DNA methylation and miR-223 (Panzade et al., 2024). This finding suggests its role as a candidate gene in fetal programming affecting chronic kidney disease risk, thereby validating the utility of co-expression networks in deciphering WD pathogenesis (Panzade et al., 2024).

The fusion of co-expression network analysis with machine learning has further enhanced analytical tools for complex diseases. Wu et al. exemplified this by combining weighted gene co-expression network analysis (WGCNA) with machine learning models (random forest, support vector machine, XGBoost) to identify cuproptosis-related key genes (ATP7B, NFE2L2, MTF1) in Parkinson’s disease (PD) (Wu et al., 2023). They developed a high-accuracy diagnostic model (AUC = 0.917) and screened potential targeted drugs (e.g., LAGASCATRIOL) via molecular docking (Wu et al., 2023). This integrated approach provides a paradigm of “co-expression network-driven module mining–machine learning-based predictive modeling–virtual screening for candidate drugs” directly applicable to WD research (Wu et al., 2023).

Notably, cuproptosis, a newly proposed cell death pathway linked to ATP7B dysfunction, may drive WD pathogenesis. (Tsvetkov et al., 2022). Multi-omics studies demonstrate that ATP7B-associated cuproptosis genes (e.g., DLAT) show aberrant expression and methylation across diseases, with prognostic implications in cancer (Liu and Tang, 2022). Critically in WD, ATP7B mutations disrupt mitochondrial metabolism (e.g., TCA cycle), activating cuproptosis and driving tissue injury, which may offer novel targets for interventions restoring copper homeostasis (Tsang et al., 2020; Davis et al., 2020). In summary, multi-omics integrative analysis, through the consolidation of multidimensional data and regulatory networks, not only provides a framework that deepens understanding of the central role of the ATP7B pathway in WD but is also poised to accelerate translational research from mechanistic insights to precision interventions, thereby paving new avenues for personalized diagnosis and treatment of WD.

### 5.2 Computational design of allosteric modulators and pharmacological chaperones

ATP7B, a Wilson’s disease-associated transmembrane copper pump, presents significant research challenges owing to its structural complexity—characterized by multiple domains and intricate molecular dynamics (Huang et al., 2014; Zhu et al., 2025). Critically, studies indicate that modulating these structural dynamics offers novel therapeutic entry points (Zhu et al., 2025; Stremmel and Weiskirchen, 2021). Huang et al. demonstrated that the N-terminal MBD1–3 of ATP7B forms a dynamic structural module through transient interactions, participating in trafficking regulation (Huang et al., 2014). Their nanobody binding experiments further revealed that targeting MBD3 (e.g., with nanobody 2R50) disrupts these transient interdomain interactions, consequently enhancing relocalization of endogenous ATP7B to the plasma membrane at the cellular level (Huang et al., 2014). Mechanistically, MD simulations revealed that this disruption allows nanobodies to recapitulate the activating effect of the native chaperone Atox1. For instance, the engineered nanobody 3MBD10 mimics Atox1 via entropy-driven binding thermodynamics, enabling the precise decoupling of regulatory activation from copper transfer (Uhlemann et al., 2020). This mechanism gains additional support from studies of other nanobodies: although binding distinct sites on MBD4 (e.g., 2R21, 1R1, 4A19, 5A51), these similarly perturb N-terminal interdomain interactions and influence ATP7B localization (Huang et al., 2014). Importantly, Huang et al. established that nanobody-mediated modulation of N-terminal domain interactions regulates ATP7B trafficking independently of copper elevation, providing direct evidence for structural dynamics-based regulation.

Expanding these concepts to pharmacological approaches, MD simulations have been successfully employed to screen and optimize small-molecule pharmacological chaperones (Kumari et al., 2018). Kumari et al. identified novel ATP7B mutations in Wilson’s disease patients that induce protein folding instability (Kumari et al., 2018). The clinically approved chaperone 4-phenylbutyrate (4-PBA) partially restores copper transport function in such destabilized mutants (van den Berghe et al., 2009), with subsequent MD studies elucidating the mechanistic basis: 4-PBA corrects folding defects by stabilizing transmembrane hydrogen-bond networks, while natural compound curcumin enhances interdomain hydrophobic interactions to compensate for reduced stability (van den Berghe et al., 2009). Collectively, these findings demonstrate how computer-aided chaperone design can guide discovery of novel stabilizing compounds, thereby enhancing druggability of allosteric targets.

Beyond conventional allosteric modulation, disrupting pathogenic protein interactions represents an emerging therapeutic strategy. Computational studies have identified key interfaces amenable to intervention. Pantoom et al. discovered a conserved LC3-interacting region (LIR3 motif) within the ATP7B C-terminus (Pantoom et al., 2021). Molecular docking simulations (GalaxyWEB) demonstrated high-affinity binding to LC3B via hydrophobic contacts at W1452/L1455, which was validated by impaired autophagosome recruitment in LIR3-mutant hepatocytes (Pantoom et al., 2021). This interface offers a druggable target for peptide mimetics or small molecules to restore autophagic flux in WD.

Beyond Wilson’s disease, ATP7B plays a significant role in resistance mechanisms to platinum-based anticancer drugs, suggesting novel strategies for modulating chemotherapy through allosteric interventions (Leonhardt et al., 2009). Leonhardt et al. demonstrated that cisplatin binds ATP7B and promotes catalytic phosphorylation at half-maximal concentrations comparable to copper (Leonhardt et al., 2009). Intriguingly, while cisplatin neither utilizes copper transport pathways nor induces ATP7B relocalization, its action strictly requires intact N-terminal MBDs—removal of the first four domains abolishes activation (Leonhardt et al., 2009). These observations suggest a unique regulatory mechanism whereby cisplatin binding perturbs cellular copper homeostasis to promote tumor resistance.

As reviewed by Lai et al., the high-affinity copper transporter Ctr1, copper chaperone Atox1, and copper efflux pumps ATP7A/ATP7B collectively regulate cellular uptake and export of platinum drugs, thereby influencing chemosensitivity (Lai et al., 2018). For instance, combining copper chelators with platinum agents enhances Ctr1 expression by reducing intracellular bioavailable copper—a strategy validated in clinical studies to overcome platinum resistance (Lai et al., 2018). This evidence provides a rationale for designing allosteric inhibitors or agonists targeting copper exporters like ATP7B to modulate platinum drug accumulation and efficacy.

Most recently, ATP7B has emerged as a potential target in tumor cuproptosis pathways (Vo et al., 2024). Luo et al. highlighted dysregulated expression of copper homeostasis regulators (including ATP7B) in lung cancer cells, correlating with copper accumulation and programmed cell death (Luo et al., 2025). Pharmacological promotion of copper influx (e.g., via elesclomol or disulfiram) or restriction of efflux (e.g., using chelators ATTM/TETA) effectively induces cuproptosis for tumor elimination (Luo et al., 2025), suggesting that small molecules modulating ATP7B’s allosteric conformations—and thereby controlling copper flux—represent promising anticancer strategies.

The successful identification of these targets underscores that computational methods leveraging ATP7B’s structural dynamics—including MD simulations and virtual screening—will serve as critical tools to identify latent allosteric sitesWhile current studies have focused on individual compounds, we anticipate that future interventions will increasingly leverage multi-scale simulations—integrating QM/MM to optimize copper transfer pathways and AlphaFold-MD pipelines—to achieve truly personalized structural editing of ATP7B.

## 6 Conclusion and future perspectives

Wilson disease, characterized by its profound genotypic diversity and complex pathophysiology, exemplifies a disorder whose understanding necessitates integration across scales. As this review highlights, computational biology provides the connective tissue: MD simulations uncover atomic-scale defects in ATP7B; ML models translate these defects into phenotypic predictions; and multi-omics networks contextualize them within systemic regulatory landscapes. This multi-scale synergy not only accelerates mechanistic discovery but also enables the rational design of targeted therapies—from nanobodies correcting allosteric dysregulation to pharmacological chaperones rescuing folding defects.

**FIGURE 3:**
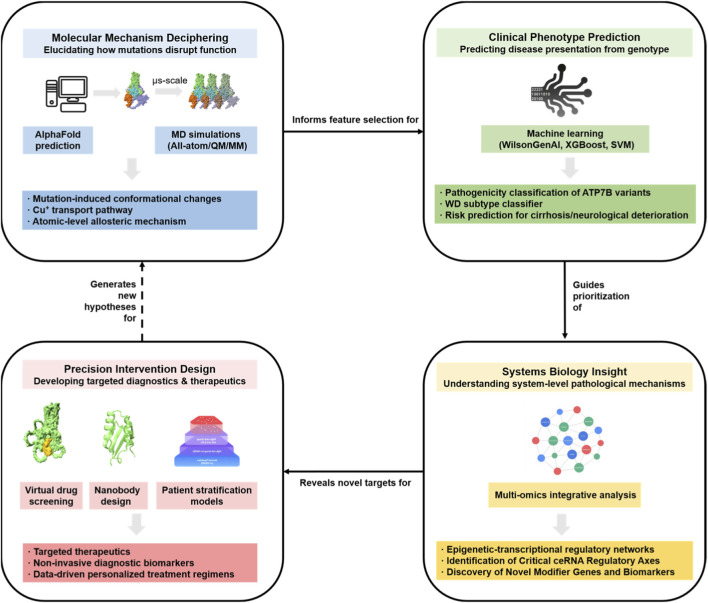
The integrative multi-scale computational framework driving precision medicine in Wilson Disease. This schematic illustrates the iterative “closed-loop” workflow proposed in this review. The pipeline begins with Molecular Mechanism Deciphering (top-left), where atomic-level structural features (e.g., misfolding dynamics) serve as mechanistic inputs that inform feature selection for Clinical Phenotype Prediction models (top-right). These predictions subsequently guide the prioritization of dysregulated pathways for Systems Biology Insight (bottom-right), enabling the identification of network-level targets. Finally, these targets drive Precision Intervention Design (bottom-left), which generates new hypotheses to be tested back at the molecular level. This cycle ensures that computational insights at one scale actively refine investigations at others.

This computational paradigm shift bridges critical gaps left by conventional methods. By enabling a holistic, multi-scale understanding from Ångströms to organisms, the integrative framework depicted in Figure 3 transforms WD research from passive observation to active, mechanism-based prediction and targeted intervention. This multi-scale synthesis is the cornerstone for accelerating the development of personalized medicine. However, challenges remain, including the demand for computational resources, the need for continued refinement and experimental validation of models, and the complexity of fully capturing the dynamic intracellular milieu and multi-organ crosstalk.

Looking beyond current capabilities, several emerging technologies hold speculative but transformative potential for WD research. Quantum computing, for instance, is theoretically poised to boost biomolecular simulations (Cordier et al., 2022), which could enable near-exhaustive modeling of ATP7B and copper-binding chemistry. Likewise, integrating single-cell multi-omics datasets will allow unprecedented resolution of the tissue- and cell-specific variations in copper metabolism (Li Y. et al., 2021), further refining systems-level models. In parallel, although still in nascent stages, AI-driven personalized medicine approaches—such as “digital twin” simulations—represent a promising frontier that may eventually guide individualized treatment strategies for WD.” In conclusion, by continuously evolving and harmonizing these computational methods, the field can expect ever deeper mechanistic insights into Wilson disease and more precise, targeted therapeutic interventions in the years ahead.
